# The complete mitogenomes of the spinyhead blenny, *Acanthemblemaria spinosa* (chaenopsidae) and the lofty triplefin, *Enneanectes altivelis* (Tripterygiidae)

**DOI:** 10.1080/23802359.2022.2034542

**Published:** 2022-02-07

**Authors:** Megan A. Sporre, Ron I. Eytan

**Affiliations:** Department of Marine Biology, Texas A&M University at Galveston, Galveston, TX, USA

**Keywords:** Chaenopsidae, Ovalentaria, cryptobenthic fish, protein-coding genes, phylogeny

## Abstract

The blennies, *Acanthemblemaria spinosa* (Chaenopsidae) and *Enneanectes altivelis* (Tripterygiidae) are representative members of two families spanning the deepest node of the Blennioidei tree. The mitogenomes of 16,507 bp for *A. spinosa* and 16,529 bp for *E. altivelis* each consisted of 37 genes and one control loop region. Phylogenetic analysis confirmed the placement of Chaenopsidae and Tripterygiidae within the Blenniiformes, however, there was instability in the placement of the triplefins between reconstruction methods, likely due to low taxon sampling. These mitogenomes represent an important milestone in uncovering relationships within Blenniiformes and Ovalentaria.

*Acanthemblemaria spinosa* (Metzelaar, 1919) is a tube blenny from the Family Chaenopsidae, and *Enneanectes altivelis* (Rosenblatt, 1960) is a triplefin blenny from the Family Tripterygiidae. Both are found throughout coral reefs in the Caribbean Sea and Bahamas and represent the families that span the deepest nodes of the Blennioidei (Wainwright et al. 2012). The mitochondrial (*COI*) substitution rate for *A. spinosa* has been estimated at 11.22% per million years (Eytan and Hellberg 2010), representing one of the fastest known vertebrate mitochondrial substitution rates (Nabholz et al. 2008, 2009; Allio et al. 2017). This, coupled with a high mitochondrial to nuclear substitution rate (37.6:1) may predispose *A. spinosa* to post-zygotic isolation through mitonuclear discordance (Eytan and Hellberg 2010; Burton et al. 2013; Lima et al. 2019). Analysis of the complete mitochondrial genome of both species furthers understanding of mitonuclear evolution and is important for the resolution of phylogenetic relationships within the Blenniiformes and Ovalentaria.

The complete mitogenomes presented here are from an *A. spinosa* individual collected from Curaçao (12.12208 N, −68.96851 W) and an *E. altivelis* individual collected from New Providence, Bahamas (25.00719 N, −77.54846 W), both are stored in 95% ethanol at the Yale Peabody Museum of Natural History (YPM ICH 23707 and YPM ICH 23717, respectively; Gregory Watkins-Colwell, gregory.watkins-colwell@yale.edu). Animal collection and handling were done in accordance with IACUC permit 2014-0133. The DNA was extracted from lateral muscle using a Qiagen DNeasy Extraction Kit and used for whole-genome sequencing of 150 bp paired-end reads on an Illumina Hi-Seq 4000 at Texas A&M University’s AgriLife genomics core facility. MITOBim (Hahn et al. 2013) was used for mitogenome assembly.

Genome annotation was performed using MitoAnnotator (Iwasaki et al. 2013) and checked using Geneious 9.0.5 (https://www.geneious.com). The 12 PCGs on the heavy strand of the mitochondrial genome of *A. spinosa*, *E. altivelis*, and 18 other species from within the Ovalentaria available on GenBank, were aligned individually using MAFFT (Katoh and Standley 2013). The 12 nucleotide alignments were concatenated into a single alignment for phylogenetic reconstruction using Bayesian (BEAST 2.6.3; Bouckaert et al. 2019) and Maximum Likelihood (IQ-TREE; Nguyen et al. 2015) methods.

The circular mitogenomes of *A. spinosa* and *E. altivelis* were 16,507 bp (GenBank Accession: MZ315025) and 16,529 bp (GenBank Accession: MZ365315), respectively. The mitogenomes are composed of 23% A, 30% C, 19.2% G, and 27.8% T bases for *A. spinosa* and 26% A, 28.5% C, 17.2% G, and 28.3% T bases for *E. altivelis*, both exhibit AT bias (49.2% GC, *A. spinosa*; 45.7% GC, *E. altivelis*). AT bias has been found in numerous other fish mitochondrial genomes (Satoh et al. 2016). The mitogenomes of both species consisted of 13 protein-coding genes, two rRNA genes, 22 tRNA genes, and one D-loop control region. Only *ND6* and eight tRNAs are found on the complementary strand. The gene order is the same as the typical vertebrate mitochondrial genome. In *A. spinosa* and *E. altivelis*, the 12S rRNA genes are 944 and 950 bp, the 16S rRNA genes are 1670 and 1690 bp, and the D-Loops are 892 and 862 bp, respectively.

In *A. spinosa*, 10 protein-coding genes use the start codon ATG, while the remaining three genes use the start codon GTG. In *E. altivelis*, eleven PCGs use the start codon ATG and two use the start codon GTG. Three genes in *A. spinosa* and two genes in *E. altivelis* share the stop codon TAG, two genes in *A. spinosa* and four genes in *E. altivelis* share the stop codon TAA, and the *ND4* gene in *A. spinosa* has the stop codon AGG. The remaining seven genes in both species have incomplete stop codons but are followed by an encoded tRNA gene or another PCG, on the same strand that may allow transcription to terminate without a complete stop codon (Pereira 2000; Satoh et al. 2016).

Both reconstruction methods recovered the Blenniiformes as a monophyletic group with Gobiesocidae as sister to the Blennioidei (Wainwright et al. 2012; Eytan et al. 2015). However, there was inconsistency in the placement of the Tripterygiidae; in the ML reconstruction Tripterygiidae and Chaenopsidae are sisters to each other and in the Bayesian reconstruction Tripterygiidae are sisters to the other Blenniiformes (Figure 1). These mitochondrial genomes represent the first complete mitochondrial genome reported from both Chaenopsidae and Tripterygiidae.

**Figure 1.**
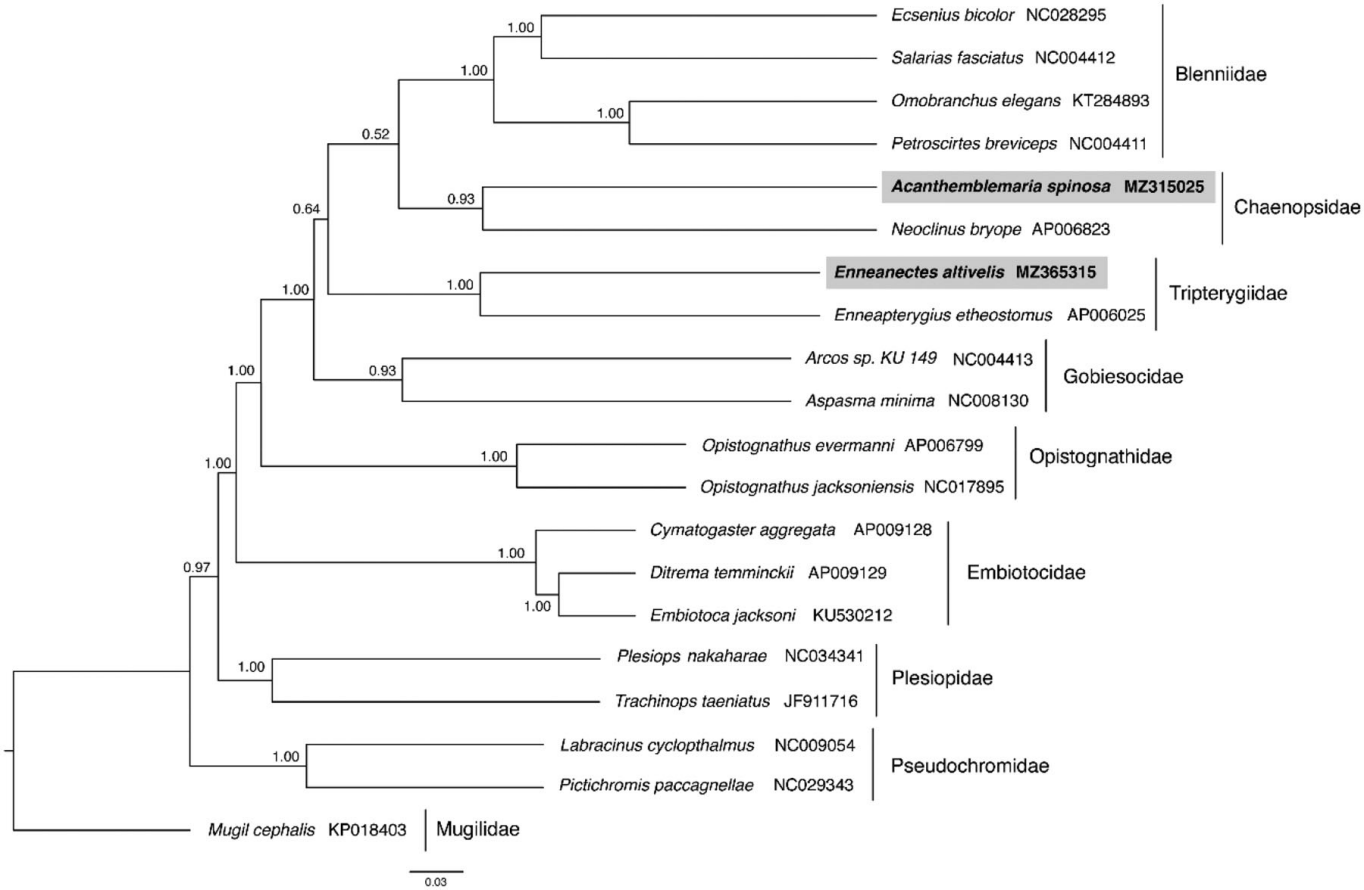
Phylogenetic tree of *Acanthemblemaria spinosa*, *Enneanectes altivelis* and 18 related species within the Ovalentaria based on 12 concatenated mitochondrial protein coding genes using Bayesian Inference (BI) methods. Nodes are labeled with the BI posterior probabilities. The concatenated PCG sequences of *Mugil cephalus* (KP018403) were used as an outgroup.

## Data Availability

Raw reads and metadata for *Acanthemblemaria spinosa* can be found under the BioProject: PRJNA785178 (BioSample: SAMN23551158; SRA: SRR17173289) and mitogenomic sequence data can be found in GenBank under the accession no. MZ315025. Raw reads and metadata for *Enneanectes altivelis* can be found under the BioProject: PRJNA785173 (BioSample: SAMN23551083; SRA: SRR17171571) and mitogenomic sequence data can be found in GenBank under the accession no. MZ365315.
